# Range expansion decreases the reproductive fitness of *Gentiana officinalis* (*Gentianaceae*)

**DOI:** 10.1038/s41598-022-06406-1

**Published:** 2022-02-14

**Authors:** Qin-zheng Hou, Nasib ur Rahman, Ahmad Ali, Yu-pei Wang, Sakhawat Shah, Ehmet Nurbiye, Wen-juan Shao, Muhammad Ilyas, Kun Sun, Rui Li, Fazal Said, Shah Fahad

**Affiliations:** 1 College of Life Science, Northwest Normal University, Lanzhou, 730070 Gansu People’s Republic of China; 2 National Key Laboratory of Crop Genetic Improvement, Huazhong Agricultural University, Wuhan, 430070 People’s Republic of China; 3 Gansu Provincial Maternity and Child-Care Hospital, Lanzhou, 730000 Gansu People’s Republic of China; 4 Hubei Insect Resources Utilization and Sustainable Pest Management Key Laboratory, College of Plant Science and Technology, Huazhong Agricultural University, Wuhan, 430070 Hubei People’s Republic of China; 5 College of Horticulture and Forestry Sciences/Hubei Engineering Technology Research Centre for Forestry Information, Huazhong Agricultural University, Wuhan, Hubei People’s Republic of China; 6 Department of Agriculture, Abdul Wali Khan University Mardan, Mardan, Pakistan; 7 Hainan Key Laboratory for Sustainable Utilization of Tropical Bioresources, College of Tropical Crops, Hainan University, Haikou, 570228 Hainan People’s Republic of China; 8 Department of Agronomy, The University of Haripur, Haripur, 22620 Khyber Pakhtunkhwa Pakistan

**Keywords:** Ecology, Plant sciences

## Abstract

Plants living at the edge of their range boundary tend to suffer an overall decline in their fitness, including growth and reproduction. However, the reproductive performance of plants in artificially expanded habitats is rarely investigated, although this type of study would provide a better understanding of range limitations and improved conservation of ex situ plants. In the current study, we transplanted a narrowly dispersed species of *Gentiana officinalis* H. Smith (Gentianaceae) from its natural area of distribution to two different elevations and natural elevation to comprehensively study its pollination biology, including flowering phenology and duration, floral display, reproductive allocation, pollinator activity, and seed production. The findings indicated that the starting point and endpoint of the flowering phenology of *G. officinalis* were earlier at the low elevation, but the peak flowering periods did not differ significantly between any of the experimental plots. When transplanted, the flowering duration, especially the female phase, was reduced; the floral display, including spray numbers, flower numbers, and flower size (length and width), decreased, especially at high elevations. Moreover, the pollen numbers and pollen-ovule ratio were decreased at both high and low elevations, although the ovule numbers showed no change, and aboveground reproductive allocation was decreased. Furthermore, pollinator richness and activity were significantly decreased, and the seed-set ratio decreased under both natural conditions and with supplemental pollination. Finally, more severe pollen limitation was found in transplanted individuals. These results indicated an overall decrease in reproductive fitness in plants living outside their original area of distribution when the geographical range of *G. officinalis* was expanded.

## Introduction

The range limitation of plant species has been a focal point in the study of ecology and evolution since Darwinian times, i.e., why species have different ranges of distribution. Knowing the governing factors of species' geographical ranges is of immense importance for predicting potential range shifts triggered by environmental changes^1^ and is also essential for the prediction of future changes in the distribution patterns. The range is partially limited by the species’ tolerance to extrinsic environmental conditions such as climate and habitat; however, other factors (e.g., life-history traits) are also involved in determining the range limits^2^. Understanding the range limits of a particular species, the causes, and the consequences are the major issues in ecological studies^3^. The interaction of species with climate, biotic and abiotic factors, competitive exclusion, and genetic bases (allelic effects and gene swamping) plays vital roles in limiting the distribution range^4,5^.

Biotic and abiotic factors, including climate change, threaten agricultural production, food security, and health. Environmental favourability declines at the peripheral points of the species range^6^. Therefore, the communities at the edge are more prone to intensive abiotic and biotic pressures. These pressures affect metabolic performance, survival, geographic distribution, interspecies competition and interactions, which limit species habitat boundaries^7–9^. Reproductive processes in plants are more vulnerable to environmental changes and ecosystem alterations, particularly sexual reproduction phases, and significant alterations have been reported in phenotypic plasticity (nongenetic responses) and evolution (genetic responses) under elevated temperatures^10,11^.

Plant populations experience novel biotic and abiotic pressures of varying magnitudes and durations^1^. Plant populations experience novel abiotic conditions upon shifting their geographical distribution, which plants did not experience earlier. Plant species experience these abiotic conditions either in duration, magnitude or in combination in a new range, which leads to a decrease in population size/density, genetic diversity, and the outcrossing rate, an increase in homozygosity within the population, and adaptive evolution of traits under new ecological conditions^11,12^. However, regardless of the negative impact expected by the reduction in genetic diversity, some introduced species perform successfully in the new distribution ranges^13^. In novel or unfavourable environments, flowering plants show reduced reproductive fitness and low reproductive output^14,15^. In *Datura stramonium*, flowers exhibited reductions in herkogamy and pollinator visits in the nonnative range compared to the native environment^13^. Moreover, *Ferula jaeschkeana* exhibited variable responses in different native and invasive environments with favourable performance. The reproductive success of the plant species varied along the altitudinal gradient. Increasing altitude resulted in a decrease in the allocation of biomass to reproductive structures in the form of decreasing dry weight, while lower altitude resulted in better reductive fitness^16^.

Here, we describe the pollination biology of a narrowly distributed plant species, *Gentiana officinalis* H. Smith (Gentianaceae), by transplanting it from its natural habitat to two artificial plots (one at a higher elevation and one at a lower elevation than the natural plot). *G. officinal* belongs to the Gentianaceae family are majorly inhibiting the temperate zones and highland regions^17^. The Tibetan Plateau is endemic for different Gentiana species including *G. officinalis*^18^. This species is widely distributed in Gansu, Qinghai and Sichuan growing on the meadows, hillside grassland and flood land of mountain at an elevation of 2300-4200 m^19^. However, being endemic to and distributed in the Qinghai-Tibetan plateau (QTP) the G. officinalis is considered a narrowly distributed specie^19,20^.The plants are 15–35 cm tall, with inflorescences crowded into terminal clusters and rare axillary whorls carrying limited flowers. The corolla is pale yellow, and individual flowers are 1.5–2.5 cm in length, with an erect, funnel-shaped corolla with five connate petals. The gynoecium includes a single bicarpellate pistil containing an ovary, a stigma and a trace of style. The ovary has parietal placentation bearing numerous lines of ovules. Five floral nectaries are situated around the base of the ovary.

We aimed to evaluate the effects of the novel environment on reproductive biology, the fitness of *G. officinalis*, and its impact on species expansion. Therefore, we investigated changes in the reproductive process, including differences in pollinators, floral characteristics, reproductive allocation, and seed production, in the transplanted populations compared with the nontransplanted plants grown in their natural environment. Given the theoretical possibility of a decrease in plant reproductive fitness at the range edges, we expect a decrease in the transplanted populations.

## Materials and methods

### Seed collection

Mature seeds of *G. officinalis* were collected from the natural-growing plant community at the Hezuo alpine meadow and wetland ecosystem research station of Lanzhou University on the southeast Qinghai-Tibet Plateau (lat. 34°53′ N, long. 101°53′ E, alt. 2900 m) in 2014 and grown in a nursery. Robust seedlings were selected and transplanted to the Haibei Alpine Meadow Ecosystem Research Station of the Chinese Academy of Sciences on the northeast Qinghai-Tibet Plateau (lat. 37°37′N, long. 101°19′ E, alt. 3200 m) and Datong ecological agriculture experimental station of the Northwest Institute of Plateau Biology (lat. 34°53′ N, long. 101°53′ E, alt. 2900 m). Transplantation was also performed in a natural environment (the Hezuo alpine meadow and a wetland ecosystem research station of Lanzhou University).

### Study plots and transplanting

The naturally studied population is located at the Hezuo alpine meadow and wetland ecosystem research station of Lanzhou University on the southeast Qinghai-Tibet Plateau (henceforth referred to as the natural environment (NE)), China (lat. 34°53′ N, long. 101°53′ E, alt. 2900 m). The third transplantation site was created in a natural environment and was termed “natural transplant” (NT). The average annual air temperature is 2 °C, with extremes of 11.5 °C (maximum) and –8.9 °C (minimum). The annual precipitation is approximately 550 mm, 80% of which falls in the short summer growing season between May and September. Hezuo station is dominated by *Kobresia humilis, Pedicularis kansuensis, Heteropappus altaicus, Stellera chamaejasme, Aconitum gymnandrum* and *Nepeta pratti,* which bloom at the same time as *G. officinalis*.

The higher-elevation transplanted plot was located at the Haibei Alpine Meadow Ecosystem Research Station of the Chinese Academy of Sciences on the northeast Qinghai-Tibet Plateau (henceforth referred to as the high-elevation environment (HE) (lat. 37°37′N, long. 101°19′ E, alt. 3200 m). The average annual air temperature was –1.7 °C, with extremes of 27.6 °C (maximum) and –37.1 °C (minimum). The annual precipitation ranged between 426 and 860 mm, mainly in July and August.

The lower-elevation transplanted plot was located at the Datong ecological agriculture experimental station of the Northwest Institute of Plateau Biology on the transition zone between Qinghai-Tibet Plateau and loess plateau (henceforth referred to as the low-elevation environment (LE)) (lat. 34°53′ N, long. 101°53′ E, alt. 2900 m). The average annual air temperature was 7.6 °C, with extremes of 34.6 °C (maximum) and –18.9 °C (minimum). The annual precipitation was approximately 380 mm, mainly in July and August. The study area was dominated by cultivated crops.

Robust seedlings with floral buds were selected for transplantation. The density of *G. officinalis* under the NE was approximately 1.5 plants/m^2^; therefore, we planted individuals at the same plant density in all transplanted plots. Moreover, more than 300 robust seedlings of *G. officinalis* were transplanted to each transplanted plot. The total planting area was greater than 200 m^2^ at each plot. The transplanted seedlings flowered in the summer, and we conducted our experiments during the following 2 years (2016–2017).

### Flowing phenology and flower duration

To observe flowering phenology, three 1 × 10-m areas were created within each experimental plot in 2016. In each plot, flower opening and duration were monitored and recorded every morning until all flowers withered.

At the full anthesis phase of *G. officinalis* in 2016, 10 plants from each plot were randomly selected. On each plant, two buds at the middle position of the inflorescence were selected, and the floral duration of all the selected buds was monitored and recorded. The pollen (male phase) and stigma (female phase) presentations were monitored and recorded.

### Floral display and reproductive allocation

At the full-bloom stage, 50 single plants were selected from each plot to test the inflorescence traits. Stem length (the distance from the stem base to apex) was measured by a straightedge. The number of sprays on each plant and the average flower numbers (including buds and fruits) on each spray were counted.

We selected 100–150 fully open flowers on different plants in each population to test the flower sizes at each plot. To avoid the position effect as much as possible, we did not choose terminal flowers. The length and width (diameter) of the flowers in each plot were measured by Vernier calipers. To test the sexual allocation changes in *G. officinalis* among the three plots, 30 buds on different plants in each plot were selected randomly. Then, the pollen numbers (PNs) and ovule numbers (ONs) were counted. The pollen/ovule ratios (P/O) were calculated as P/O = pollen numbers in all five anthers/ovule numbers^21^.

Sampling dates corresponded to the height of the flowering season at each site (mid-August in the LE and early September in the NE and HE) before fruiting had occurred. While fresh, the aboveground parts of 30 fully flowering plants per site were dissected into inflorescences, peduncles, leaves, and stems. Plant material was oven-dried at 70 °C for 3 days, and the dry weights were obtained to the nearest 0.1 mg on an analytical balance (Ohaus). The inflorescence and peduncle fractions of each plant were summed to provide a measure of reproductive biomass (R), and the leaf and stem fractions of each plant were summed to provide a measure of vegetative biomass (V). The reproductive allocation (RA) was calculated as RA = R/(R + V).

### Observation of pollinators

The floral visitors to *G. officinalis* were recorded in the three plots. Ten neighbouring inflorescences on different individual plants were selected at random and labelled. Before observation, we counted all the open flowers on one inflorescence and then recorded the number of flowers visited by pollinators. We observed these flowers between 9:00 a.m. and 6:00 p.m. in each plot during 2016 and 2017. In total, observations were carried out for 65 h in each plot over the 2 years. While carrying out these observations, we stayed 2 m away from the focal flowers to observe all of the floral visitors without disturbing their foraging behaviours. The visitor species, behaviour in the flower, and visiting times of each species were recorded, and the visit frequencies of each visitor species were calculated. The visit frequency was calculated as visit frequency = visit times/visit flower numbers/hour.

To identify whether flower visitors were legitimate pollinators of *G. officinalis*, collected visitors were observed and photographed with a stereomicroscope to identify whether *G. officinalis* pollen was attached to their bodies. Additionally, each visitor was observed to determine whether the reproductive structures of flowers had been touched. Visitors that were positive for all these factors were considered legitimate pollinators.

### Seed production

To test the self-compatibility of *G. officinalis*, flowers subjected to self-pollination treatment (unopened flowers were isolated with paper bags) in 2017 on the three plots were subjected. To further analyse self-compatibility, we conducted outcrossing pollination. In addition, 30 individual inflorescences on different plants were bagged, and two buds at the same position on each inflorescence were selected. Both buds on each inflorescence were emasculated before the flowers opened. When the stigma opened, one flower was pollinated with fresh pollen from the same inflorescence or different inflorescences on the same plant (selfing), and the other was pollinated with fresh pollen from a plant 5 m away (outcrossing). To test whether facilitated selfing occurred, 30 individual plants in each plot were tagged. On each tagged plant, two individual buds were selected: one was assigned to natural pollination, and the other was assigned to emasculation (removal of all anthers before stigma lobe opening). To test whether agamospermy occurred, the flowers were subjected to emasculation treatment and isolated in three plots. Thirty buds on different plants were randomly selected, and all the anthers were removed before the flowers opened, and then all the buds were isolated with paper bags. At maturity, all fruits were collected, and all of the seeds (including mature and abortive seeds) were counted. Seed-set ratios were used to assess the reproductive success of each treatment, which were calculated by the number of mature seeds divided by the total ovules in each ovary. The facilitated selfing data were calculated as the natural seed-set ratio minus the emasculated seed-set ratio.

Similarly, 30 inflorescences were tagged on different plants in each plot, and two buds were then tagged at the same position on each inflorescence; one bud was assigned to natural pollination, and the other was assigned to supplemental hand pollination when stigmas opened. For supplemental hand pollination, pollen was collected randomly from unmarked individuals at a minimum distance of 5 m from the recipient individual. Supplemental hand pollination events were conducted every day until the flower was permanently closed. When mature, all seeds were counted, and seed-set ratios were calculated. For each plot, we calculated an index of pollen limitation (IPL): IPL = 1 − (Po/Ps), where Po is the natural seed-set ratio and Ps is the supplemental hand-pollination seed-set ratio. As the seed-set ratios showed no significant difference between natural and supplemental hand pollination in the natural environment, we considered the IPL at this plot to be 0. The IPL data at the other two plots were compared using an independent-samples t test.

### Statistical analysis

The normality of the data was tested using one-sample Kolmogorov–Smirnov (1-K-S) tests, and then one-way ANOVAs (with Tukey's multiple contrasts) were used to test differences in all traits among the three environments.

## Results

### Range expansion affects flowering phenology and flower duration

**Figure 1:**
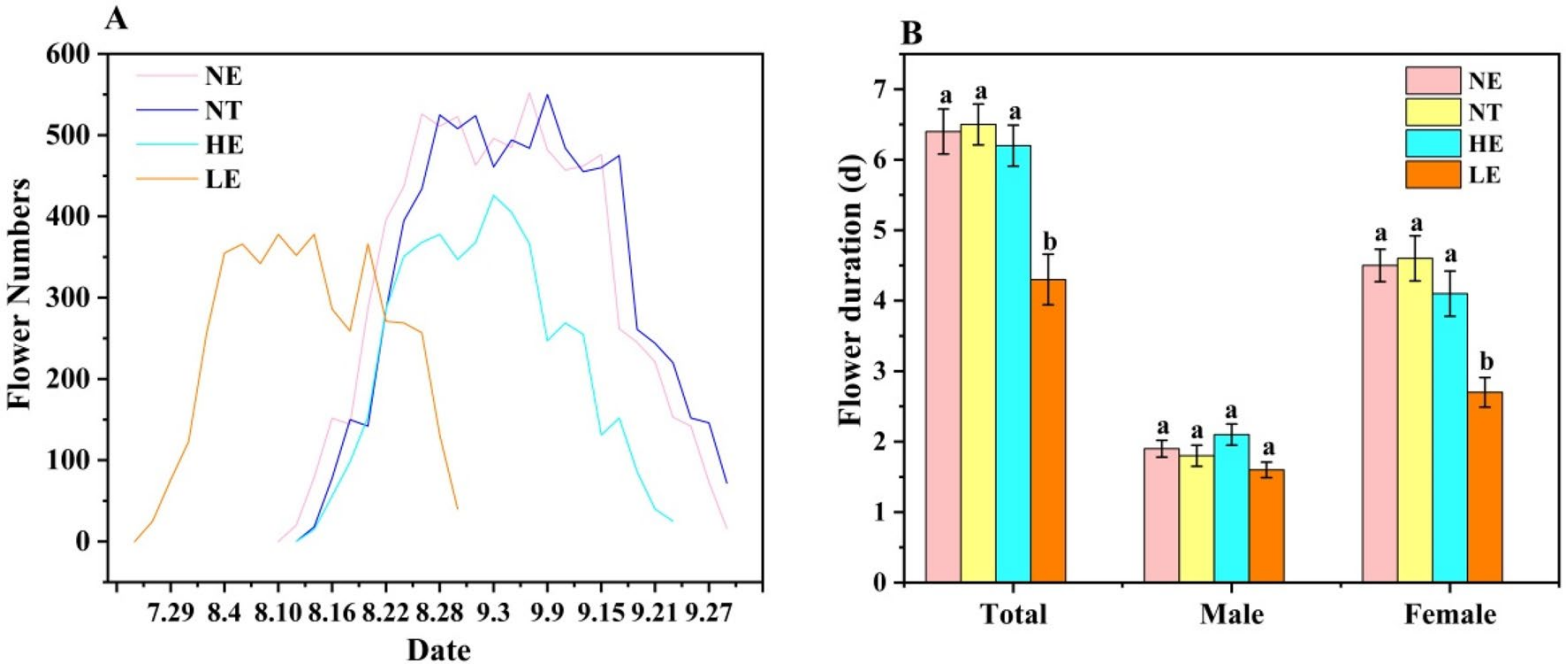
Effects of range expansion on the flowering phenology and duration of *G. officinalis*. (**A**) represents the flowering phenology of *G. officinalis* in four different plots belonging to NE, NT, LE, and HE. All the opened flowers were monitored and recorded in each plot every morning until all the flowers withered. (**B**) shows the male and female flower duration of *G. officinalis* in the abovementioned plots.

The flowering phenology of *G. officinalis* varied significantly among the three plots set at NE , HE , LE and transplantation in the NE (NT) as a control. *G. officinalis* flowering began in early August in the NE, which was the same as in the HE, but much later (15 d) than in the LE (late July). The phenology end date of *G. officinalis* at the NE was late September, which was the same as the HE but much later (30 d) than the LE (late August). The phenology of *G. officinalis* at the NE lasted approximately 50 days, which was longer than that at the HE (40 d) and LE (30 d). The peak flowering period of G. officinalis at the NE lasting approximately 20 d (from early August to mid-August) was the same as that in the two transplanted environments (Fig. 1A).

At the NE, the total flowering duration of *G. officinalis* lasted for 6.3 ± 0.5 d, which was longer than that of the two transplanted environments (4.6 ± 0.4 d at the HE and 4.5 ± 0.6 d at the LE). The male phase durations of *G. officinalis* did not differ significantly among the three plots, but the female phase duration at the NE (4.2 ± 0.3 d) was much longer than that for the other two plots (2.9 ± 0.2 d at the HE and 2.7 ± 0.5 d at the LE) (Fig. 1B).

To ensure that this phenomenon was not due to the transplantation of natural population, the flower phenology and duration were compared with those of transplantation at natural elevation (NT). When comparing the NT with NE conditions, no significant difference in the flowering phenology or duration was found (Fig. 1A and B). Together, these data suggest that range expansion significantly alters the flower phenology and duration of *G. officinalis*.

### *G. officinalis* changes the floral display at expanded ranges and produces more biomass at the HE

**Figure 2:**
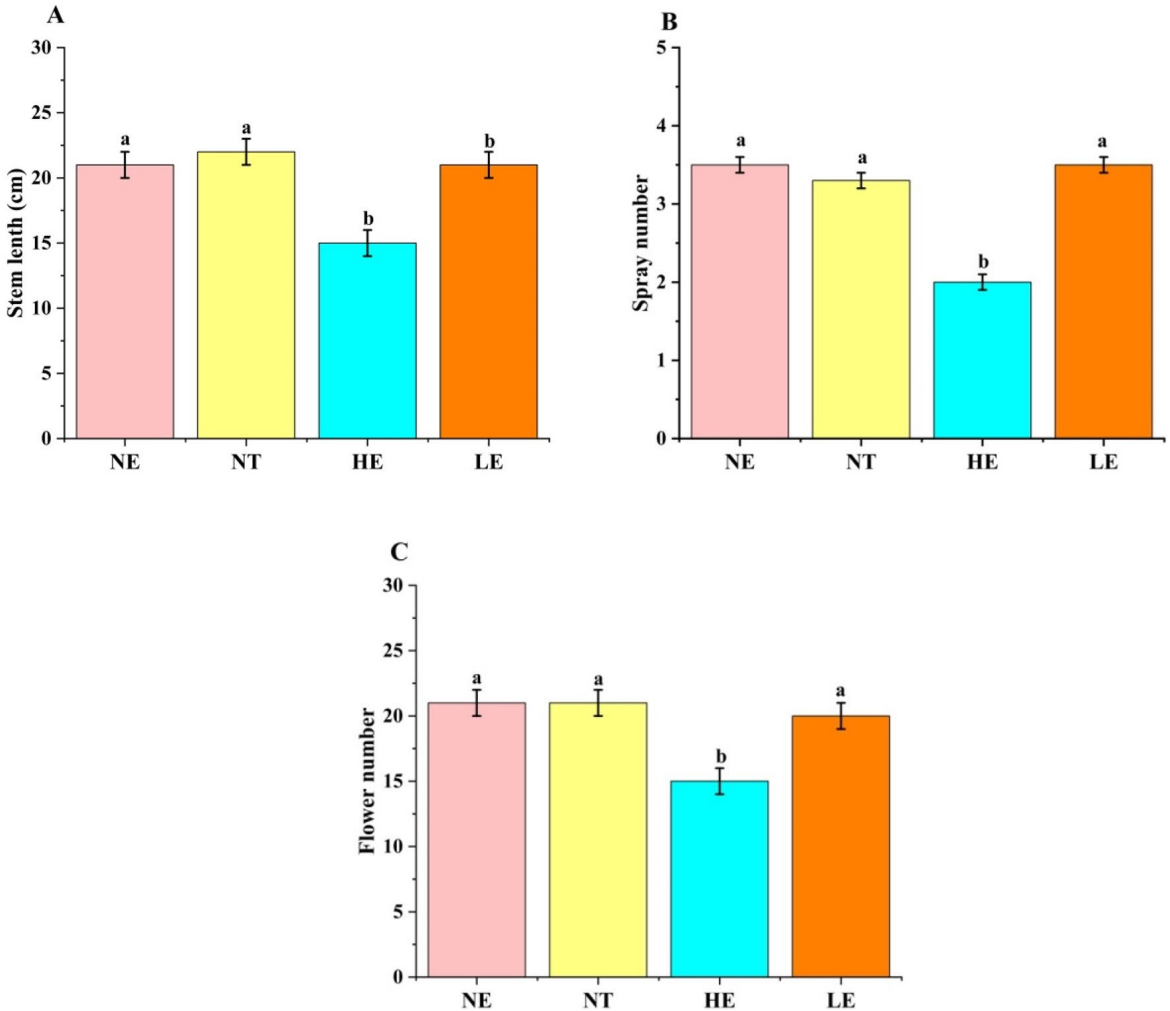
The floral display of *G. officinalis* among the 3 plots. (**A**), The stem length of each plant (distance from stem base to apex). (**B**), the number of sprays on each plant. (**C**), the average flower number on each spray.

To determine whether range expansion affects flowering features and the overall structure of *G. officinalis* flowers, we studied the total number of sprays on a single plant and the number of flowers per spray in each plot. At the HE, *G. officinalis* comprised 2.1 ± 0.5 sprays on a single plant and 13.4 ± 5.4 flowers on a single inflorescence, which was less than those at the other two plots (3.5 ± 1.5 sprays and 22.5 ± 8.1 flowers at the NE 3.7 ± 1.3 sprays and 24.5 ± 9.6 flowers at the LE); no significant difference was identified between the numbers from NE and LE sites. Similarly, the plants were shorter at the HE (stem length 15.2 ± 4.2 cm) than at the other two plots (stem length at the NE 20.9 ± 5.9 cm and LE 21.3 ± 4.8 cm) (Fig. 2A and B). No significant difference was noted between the stem lengths in the two transplanted environments (Fig. 2C).

**Figure 3:**
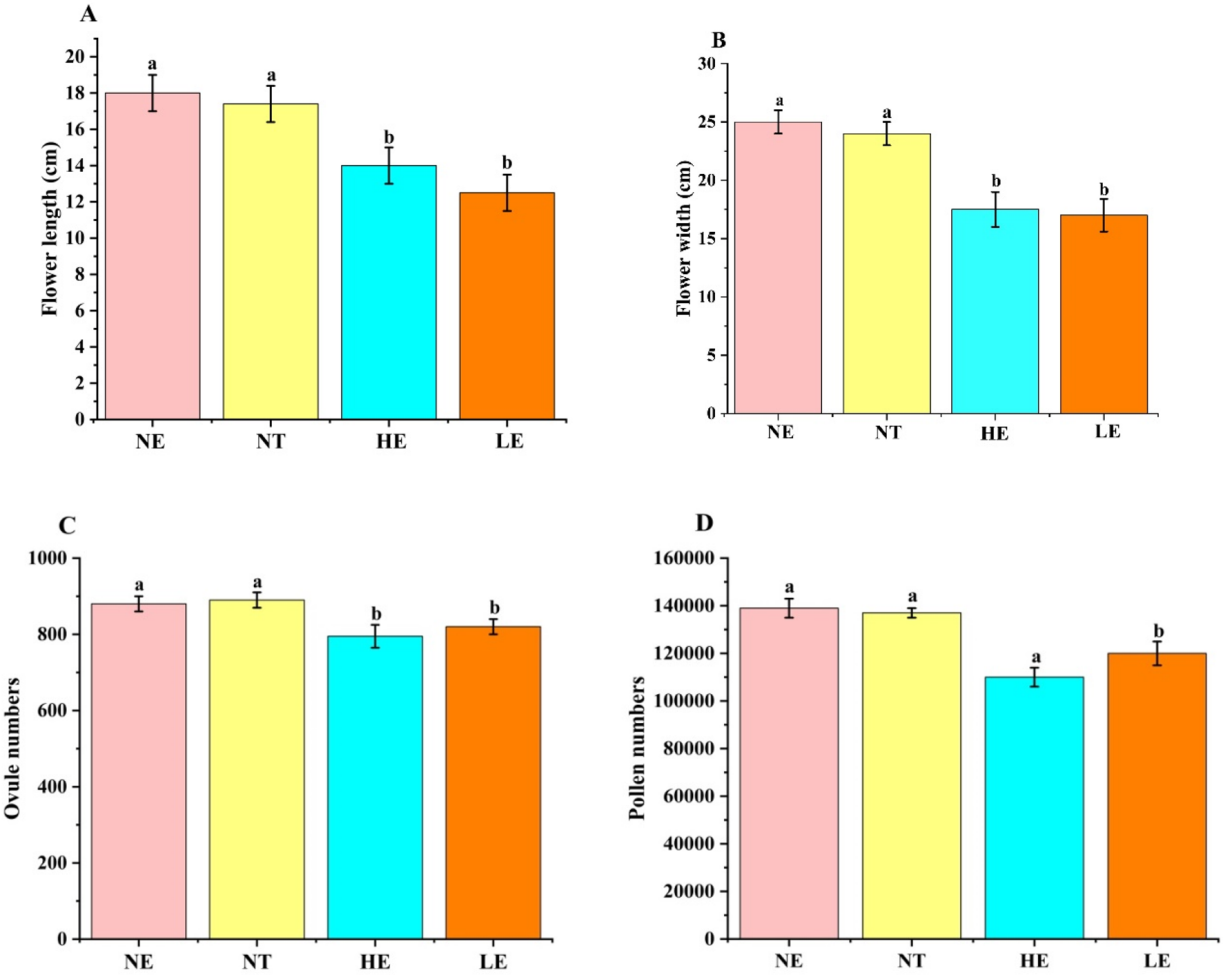
Effect of range expansion on floral and vegetative biomass and floral, ovule, and ovary numbers in *G. officinalis.* (**A**), Length of the flower (length from the top to the bottom of the flower). (**B**), width of the flower (flower diameter). (**C**), ovule numbers and (**D**), pollen numbers.

The flower size (length and width) of *G. officinalis* was smaller at both expanded ranges (HE and LE) than in the natural range (NE and NT) in terms of length (12.6 ± 1.5 and 13.7 ± 2.2 cm at the LE and HE, respectively, and 18.6 ± 1.7 cm at the NE) and width (16.7 ± 2.1 and 17.1 ± 2.8 cm at the LE and HE, respectively, and 25.3 ± 2.4 cm at the NE). Flower size showed no significant difference between HE and LE (Fig. 3A and B).

A single flower at the NE produced a similar number of ovules (155 ± 42) to flowers in the transplanted environments (144 ± 51 at the LE and 149 ± 39 at the HE) but more pollen (136,800 ± 5310) than those in the other plots (116,630 ± 5820 at the LE and 109,180 ± 6040 at the HE) (Fig. 3C and D); however, no significant difference was found between the two transplanted environments. Similarly, the P/O ratio at the NE (882 ± 25) was higher than those at the other two plots (809 ± 31 at the LE and 792 ± 29 at the HE), and no significant difference was observed between the two transplanted environments (Fig. 3).

In the NE and NT, both the aboveground vegetative biomass (V, 1.98 ± 0.12 g) and reproductive biomass (R, 1.27 ± 0.11 g) of *G. officinalis* were smaller than those at LE (2.62 ± 0.17 g and 1.69 ± 0.18 g of V and R, respectively) but higher than those at HE (1.62 ± 0.21 g and 1.04 ± 0.12 g of V and R, respectively) (Fig. 3C and D). These results demonstrated that range expansion significantly reduces the overall number of both ovules and pollen in *G. officinalis*. However, the plant produces lower biomass of both the reproductive and vegetative parts at the HE.

### Range expansion changes reproductive allocations and overall observation visitors

**Figure 4:**
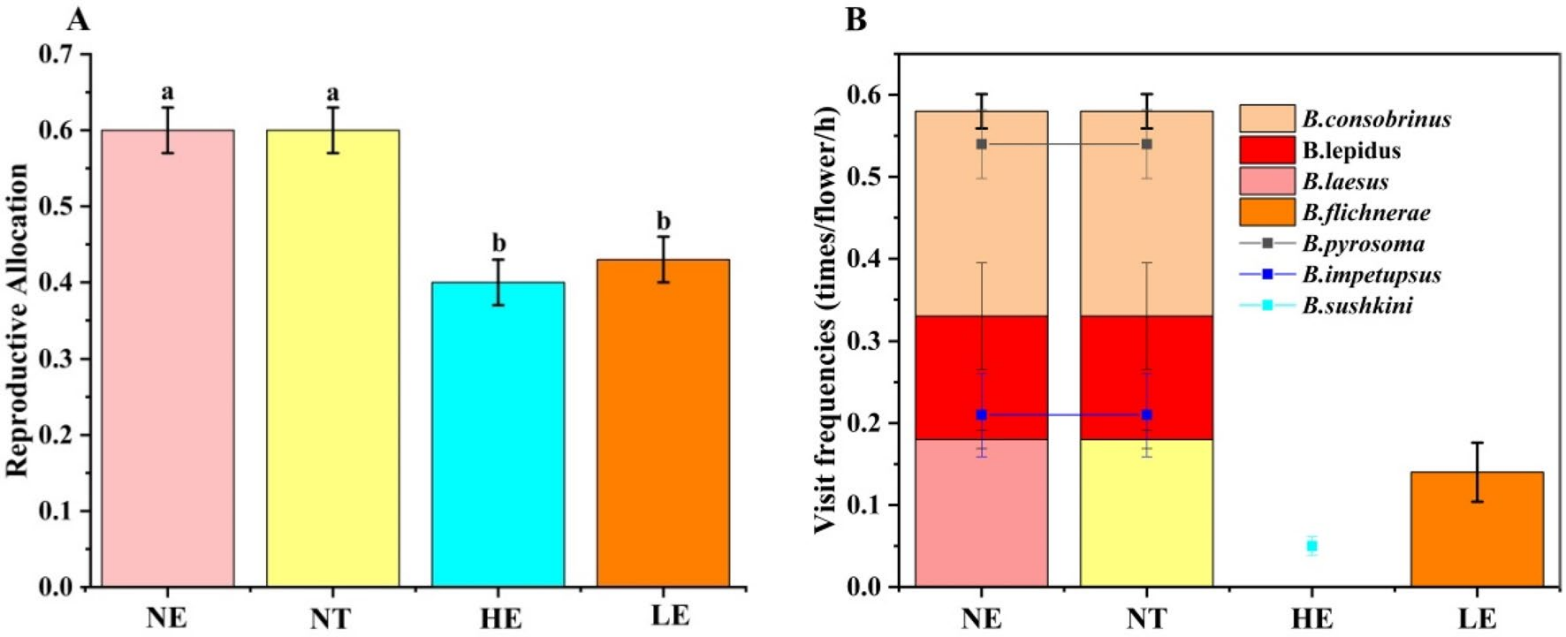
Effect of range expansion on reproductive allocations and floral visitors’ frequencies. (**A**), Reproductive allocation of *G. officinalis* among all three plots. (**B**), The floral visitors' visit frequencies among the plots. Data were collected in three plots in 2016 and 2017 for a total of 65 h of field observations**.**

The reproductive allocation for *G. officinalis* at the NE and NT (0.61 ± 0.09) was larger than that at the two transplanted environments (0.42 ± 0.09 and 0.41 ± 0.11 at the LE and HE, respectively), but no significant difference was found between the two transplanted environments (Fig. 4A), and during the 2 years of observation, we identified five pollinator species at the NE, all of which were bumblebees. The five pollinator species were *Bombus consobrinus*, *B. pyrosoma*, *B. lepidus*, *B. impetupsus*, and *B. laesus*, with visit frequencies of 0.58, 0.54, 0.33, 0.21, and 0.18 times/flower/h, respectively (Fig. 4A). Based on the pollinator visit behaviours and pollen transfer modes, the five pollinator species were identified as belonging to one pollinator functional group. Therefore, we calculated the frequency of pollinator visits to *G. officinalis* as the summation of the visit frequencies of all five pollinator species (1.84 times/flower/h) but did not calculate the visit efficiency of each pollinator.

In the transplanted environments, the pollinator assemblages and visit frequencies were significantly lower than those in the NE. The only pollinator of *G. officinalis* at the LE was B. *filchnerae,* and the visit frequency was 0.47 times/flower/h. The only pollinator of *G. officinalis* at the HE was *B. sushkini,* and the visit frequency was 0.14 times/flower/h (Fig. 4B).

Together, these results suggested that the range of expansion of *G. officinalis* significantly reduces reproductive allocations and visiting frequency of pollinators during the flowering season.

### Effect of range expansion on seed production in *G. officinalis*

**Figure 5:**
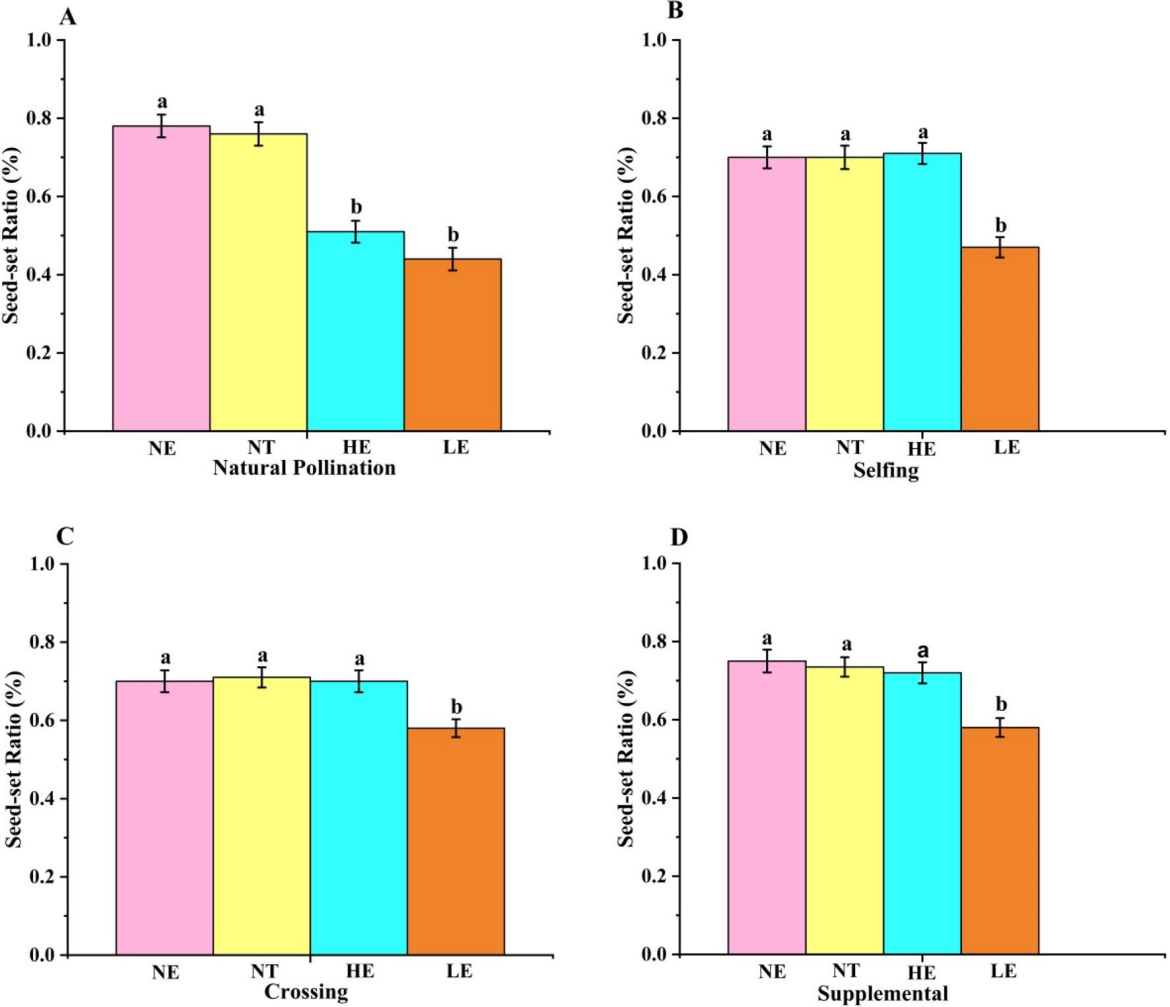
The seed-set ratio of *G. officinalis* under 3 pollination conditions among the 4 plots. (**A**) represents natural pollinator, (**B**) depicts self-pollination, (**C**) shows cross-pollination, and (**D**) represents supplemental pollination. Data were collected from 2 buds at the same position on each inflorescence (a total of 30 inflorescences) on different plants in each plot, with one bud for natural pollination and the other for supplemental hand pollination when stigmas opened until permanent flower closure.

*G. officinalis* was self-compatible in all three plots, since the selfing seed-set ratio was high and showed no significant difference compared with outcrossing (Fig. 5). Facilitated selfing occurred in all plots (0.11, 0.15, and 0.16 at the NE, LE, and HE, respectively), indicating that incomplete dichogamy may occur in *G. officinalis* and that pollinator activities in flowers may facilitate selfing. No agamospermy of *G. officinalis* occurred in the three plots, as no seeds were produced after emasculation and isolation.

Under natural pollination conditions, the seed-set ratio of *G. officinalis* was higher under NE (0.77 ± 0.11) than in the transplanted environments (0.47 ± 0.09 under LE and 0.55 ± 0.10 under HE). The seed setting in the NT was copmaraable to NE (0.76 ± 0.25). When enough pollen was deposited on the stigma (supplemental pollination), the seed-set ratio at the LE (0.59 ± 0.11) was significantly lower than that at the other two plots (0.76 ± 0.11 at the NE and 0.74 ± 0.06 at the HE) (Fig. 5). At the NE, the IPL was 0 because the natural seed-set ratio did not differ significantly with supplemental hand pollination. In the transplanted environments, the seed-set ratio after supplemental hand pollination was significantly higher than that for natural pollination, indicating that pollen limitations occurred in the two transplanted environments. The IPL values were 0.10 and 0.23 at the LE and HE, respectively; the IPL at the HE was significantly higher than that at the LE (Fig. 5).

### Ethical approval

Experimental research and field studies on plants (either cultivated or wild) comply with relevant institutional, national, and international guidelines and legislation. All plant studies (*Gentiana officinalis*) were carried out in accordance with relevant institutional, national or international guidelines or regulations.

## Discussion

During the process of invasion, plants experience various biotic and abiotic pressures (suboptimal conditions) that potentially alter the normal life processes (negative change) of the invading organism^22^ There is limited understanding of how a species responds to novel stresses in a new environment^1^. However, organisms overcome novel stresses via behavioural modifications, physiological plasticity, and acclimatization potential or local adaptation governed by natural variations^23–27^.

Reproductive fitness and reproductive potential are the fundamental determinants of individual and population success, which is affected by the novel habitat through varying environmental and biological determinants, potentially challenging the individual’s contribution to the persistence and expansion of a colonizing population^22^. In our study, the blooming period for *G. officinalis* was earlier in the low-elevation environment than in the natural and high-elevation environments. The climatic factors (higher air temperatures and dry conditions) in the low-elevation environment might be the reason for this alteration because these factors induced early flowering^28,29^. Although the phenology of *G. officinalis* was 10–20 days shorter in the transplanted environment than in the natural environment, the peak flowering period remained coincident among the three plots. These results suggest that when the geographical range is expanded, *G. officinalis* may flower in a more concentrated period. As a result, the flowering phenology may not be restricted or potentially altered by the expansion of the species’ range.

During the bloom period, flowers perform their respective reproductive functions (pollen dehiscence, stigma receptivity, pollen germination, and pollen tube growth), which are important for pollination^30^. Disturbed environments, suboptimal habitats or local conditions (reduced or no availability of pollinators) expand the floral duration for successful pollination^21^. In contrast to the pollination assurance hypothesis^31^, no significant correlation was observed between visit frequencies and floral duration in the two populations of *G. straminea*. Our results show that the pollinator assemblages and visit frequencies to *G. officinalis* were lower in the transplanted environments than in the natural environment, but the flower duration, especially the female phase duration, was not correspondingly prolonged, as suggested by the pollination assurance hypothesis. Therefore, pollinator richness and activities may not play a key role in shaping the plasticity of floral duration. Previous studies have shown that high temperature and dry conditions may shorten floral duration^32,33^. We observed that the flowering duration of *G. officinalis* was shorter under high temperatures and dry conditions (low-elevation environment), which agrees with previous viewpoints. However, we differently concluded here because we observed shortened floral duration in a high-elevation environment (relatively low temperatures and wet conditions). Based on our results, we speculate that unsuitable environmental factors, mainly air temperature and precipitation, in the transplanted environments reduced the floral duration, which in turn negatively affected plant reproductive fitness, especially female fitness.

Self-compatibility and combined dichogamy and herkogamy occurred in *G. officinalis*, as reported in other species of the *genus Gentiana*^34^. However, facilitated selfing indicates that incomplete dichogamy occurs in *G. officinalis*, which was reported earlier in *Gentiana*. Incomplete dichogamy (a period of overlap between male and female phases) may have positive or negative effects on reproductive fitness, while the overlap of sexual phases provides reproductive assurance (opportunities to produce seeds via self-pollination). However, this incomplete dichogamy may also induce selfing and inbreeding depression and reduce genetic variation^35^. The reduced genetic diversity and inbreeding depression may be involved in the narrow distribution of *G. officinalis*. However, the benefits of "reproductive assurance" favoured by incomplete dichogamy, which occurred in the transplanted environments, did not promote the wider distribution of *G. officinalis*, indicating that some other factors limited plant population expansion.

Levels of seed production (and hence seed dispersal) imply the potential for the successful establishment of new populations^36^. Many species exhibit a decline in seed production towards their range boundaries^37^. In the current study, when the geographical range of *G. officinalis* was artificially expanded, the seed-set ratios were significantly reduced, especially in the high-elevation plots. These results suggest a reduction in the total fitness of *G. officinalis* in the transplanted environments. Seed production can be affected by diverse factors that act during flower and fruit development, which are summarized as pollen limitation (factors limiting ovule fertilization) and resource limitation (factors affecting the likelihood of completing seed development)^38^. The restriction of resources is often encountered by plants under natural pollination conditions and may lead to reduced seed production^39^. Several studies have shown that resource limitations should be considered if seed production does not increase after supplemental hand pollination^40^*.* Some studies have also suggested that resources may be reallocated by plants between flowers, i.e., although supplemental hand pollination may increase the seed set, the increased seed set may be offset by a decreased seed set in other flowers^41^. As a result, multiple controls, including resource reduction and resource addition, have been included in some studies^39^. Our results showed that compared to the natural environment, the seed-set ratio after supplemental hand pollination of *G. officinalis* was much lower in the low-elevation environment but remained the same in the high-elevation environment. We did not conduct multiple controls to test whether these results were due to resource limitations in a low-elevation environment or resource shifting from less pollinated flowers to more highly pollinated flowers to support a larger seed set in the other two environments, but we concluded that seed production was hindered when the geographical range of *G. officinalis* expanded, especially to a lower elevation.

A decline in pollinator availability has strong negative effects on plant pollination because it changes plant-pollinator interactive networks and consequently impacts reproductive success due to pollen limitation^21^. Here, we report that the pollinator assemblages and visit frequencies to *G. officinalis* declined, and consequently, the IPL increased in the transplanted plots, which affirmatively supports the viewpoint that pollinator richness and activities are key factors in plant reproductive success. Floral characteristics, including flower number, flower size, and reproductive allocation, are considered the major signs of decreased pollinator richness in altered environments^21^. We showed that the floral display (flower numbers, plant height, flower sizes, and aboveground reproductive resource allocation) of *G. officinalis* was lower and that reproductive allocation was reduced in the transplanted environments, rendering the plants less attractive to pollinators. Furthermore, although the ovule numbers of *G. officinalis* remained the same in the three plots, the pollen numbers and P/O ratios were significantly reduced in the transplanted plots. The pollination efficiency hypothesis^42^ points out that the pollination efficiency index (the proportion of pollen grains removed from anthers that are subsequently deposited on conspecific stigmas) correlated negatively with the P/O ratio, i.e., a lower P/O is necessary to guarantee the maximum seed set^43^. Our results disagree with this hypothesis since the lower P/O ratio of *G. officinalis* in the transplanted environments did not result in a high seed-set ratio. However, diverse selective factors likely affect the evolution of P/O ratios, and different selection factors play a role in the different processes of reproduction^44,45^.
